# Ancient reindeer mitogenomes reveal island-hopping colonisation of the Arctic archipelagos

**DOI:** 10.1038/s41598-024-54296-2

**Published:** 2024-02-20

**Authors:** Katharina Hold, Edana Lord, Jaelle C. Brealey, Mathilde Le Moullec, Vanessa C. Bieker, Martin R. Ellegaard, Jacob A. Rasmussen, Fabian L. Kellner, Katerina Guschanski, Glenn Yannic, Knut H. Røed, Brage B. Hansen, Love Dalén, Michael D. Martin, Nicolas Dussex

**Affiliations:** 1 Department of Natural History, NTNU University Museum, Norwegian University of Science and Technology (NTNU), Erling Skakkes Gate 47B, 7012 Trondheim, Norway; 2 Centre for Palaeogenetics, Svante Arrhenius väg 20C, 10691 Stockholm, Sweden; 3 Department of Bioinformatics and Genetics, Swedish Museum of Natural History, 10405, Stockholm, Sweden; 4 Department of Zoology, Stockholm University, 10691 Stockholm, Sweden; 5 Animal Ecology, Department of Ecology and Genetics, Evolutionary Biology Centre, Uppsala University, Norbyvägen 18D, 75236, Uppsala, Sweden; 6 Institute of Ecology and Evolution, School of Biological Sciences, University of Edinburgh, Edinburgh, UK; 7 Gjærevoll Centre for Biodiversity Foresight Analyses, Norwegian University of Science and Technology (NTNU), 7491, Trondheim, Norway; 8 Department of Terrestrial Ecology, Norwegian Institute of Nature Research (NINA), Høgskoleringen 9, 7034, Trondheim, Norway; 9 Univ. Savoie Mont Blanc, CNRS, LECA, Laboratoire d’Ecologie Alpine, Univ. Grenoble Alpes, 38000 Grenoble, France; 10 Department of Preclinical Sciences and Pathology, Norwegian University of Life Sciences, P.O. Box 5003, 1432 Ås, Norway; 11 Globe Institute, University of Copenhagen, Øster Voldgade 5-7, 1350, Copenhagen, Denmark; 12 Department of Mammals and Birds, Greenland, Institute of Natural Resources, Kivioq 2, 3900 Nuuk, Greenland

**Keywords:** Evolution, Phylogenetics, Genetics, Evolutionary biology, Genomics, Haplotypes

## Abstract

Climate warming at the end of the last glacial period had profound effects on the distribution of cold-adapted species. As their range shifted towards northern latitudes, they were able to colonise previously glaciated areas, including remote Arctic islands. However, there is still uncertainty about the routes and timing of colonisation. At the end of the last ice age, reindeer/caribou (*Rangifer tarandus*) expanded to the Holarctic region and colonised the archipelagos of Svalbard and Franz Josef Land. Earlier studies have proposed two possible colonisation routes, either from the Eurasian mainland or from Canada via Greenland. Here, we used 174 ancient, historical and modern mitogenomes to reconstruct the phylogeny of reindeer across its whole range and to infer the colonisation route of the Arctic islands. Our data shows a close affinity among Svalbard, Franz Josef Land and Novaya Zemlya reindeer. We also found tentative evidence for positive selection in the mitochondrial gene ND4, which is possibly associated with increased heat production. Our results thus support a colonisation of the Eurasian Arctic archipelagos from the Eurasian mainland and provide some insights into the evolutionary history and adaptation of the species to its High Arctic habitat.

## Introduction

The repeated glacial fluctuations of the Quaternary had a major impact on the geographic distribution and the genetic structure of temperate and cold-adapted species^1,2^. Climatic and glacial oscillations forced species to move, adapt or be driven to extinction^2^. Thus species ranges repeatedly expanded and contracted as they tracked their preferred habitat^1–4^. During glacial/interglacial periods, species persisted in refugia^5,6^. The main glacial refugia in Europe were located on the Iberian and Italian peninsulas, as well as the Balkans^7^. In North America and Siberia, the main refugium was Beringia, which encompassed the Bering land bridge, parts of Yukon, Alaska and eastern Siberia^8,9^. Responding to the climatic oscillations in the opposite way than temperate species, cold-adapted species had larger distributions during glaciations and were confined to refugia during interglacials^5,6^. At the end of the last glacial maximum (LGM; ca. 18,000–20,000 years before present (BP)), cold-adapted species such as the Arctic fox (*Alopex lagopus*), ptarmigan (*Lagopus muta*), collared lemmings (*Dicrostonyx torquatus*), and reindeer/caribou (*Rangifer tarandus*) re-colonised the formerly glaciated areas of the High Arctic, including its remote islands, leading to their current circumpolar distributions in the Holarctic region^2,10–12^.

Svalbard and Franz Josef Land, each composed of hundreds of islands, are the most remote archipelagos of the Holarctic region. As both archipelagos were almost entirely glaciated during the LGM, most current plant and animal species must have re-colonised the archipelagos after the retreat of the ice sheets (i.e. 10,000–15,000 years BP), thus making them a great study system for the genetic and evolutionary effects of recent colonisation and isolation^13–15^.

*Rangifer tarandus,* referred to as reindeer in Eurasia and caribou in North America, is an ideal species with which to study the impact of glacial cycles on species distributions^16–18^. Its current distribution spreads from the High Arctic (including Svalbard) to the tundra and taiga regions of northern Europe, Siberia, North America and Greenland^2,18–21^. The species is well adapted to various environments and shows four major ecotypes: the boreal forest form, the barren-ground or tundra form, the mountain form and the arctic form^16–18,22^.

Previous studies indicate that ca. 70,000 years BP an ancestral lineage of *Rangifer tarandus* split into two distinct mitochondrial lineages that represent two different refugia: the Euro-Beringian lineage (BEL) and the North American lineage (NAL)^6,23–27^. The BEL lineage is the most diverse and considered as the ancestral lineage for *Rangifer tarandus*^25^. The NAL lineage likely originated south of the North American ice sheet, where reindeer survived before expanding northward^26^. Moreover, some BEL sublineages expanded south across North America from Beringia when the ice retreated and secondary contact occurred in Canada ca. 8000 years BP, corresponding with the final deglaciation of North America^20,23–28^. Consequently, the two lineages interbred and now occur as genetically admixed populations in Western North America. Reindeer are extinct on Franz Josef Land, but ancient remains suggest that they occupied the archipelago ca. 6400–1300 years BP^21^.

**Figure 1:**
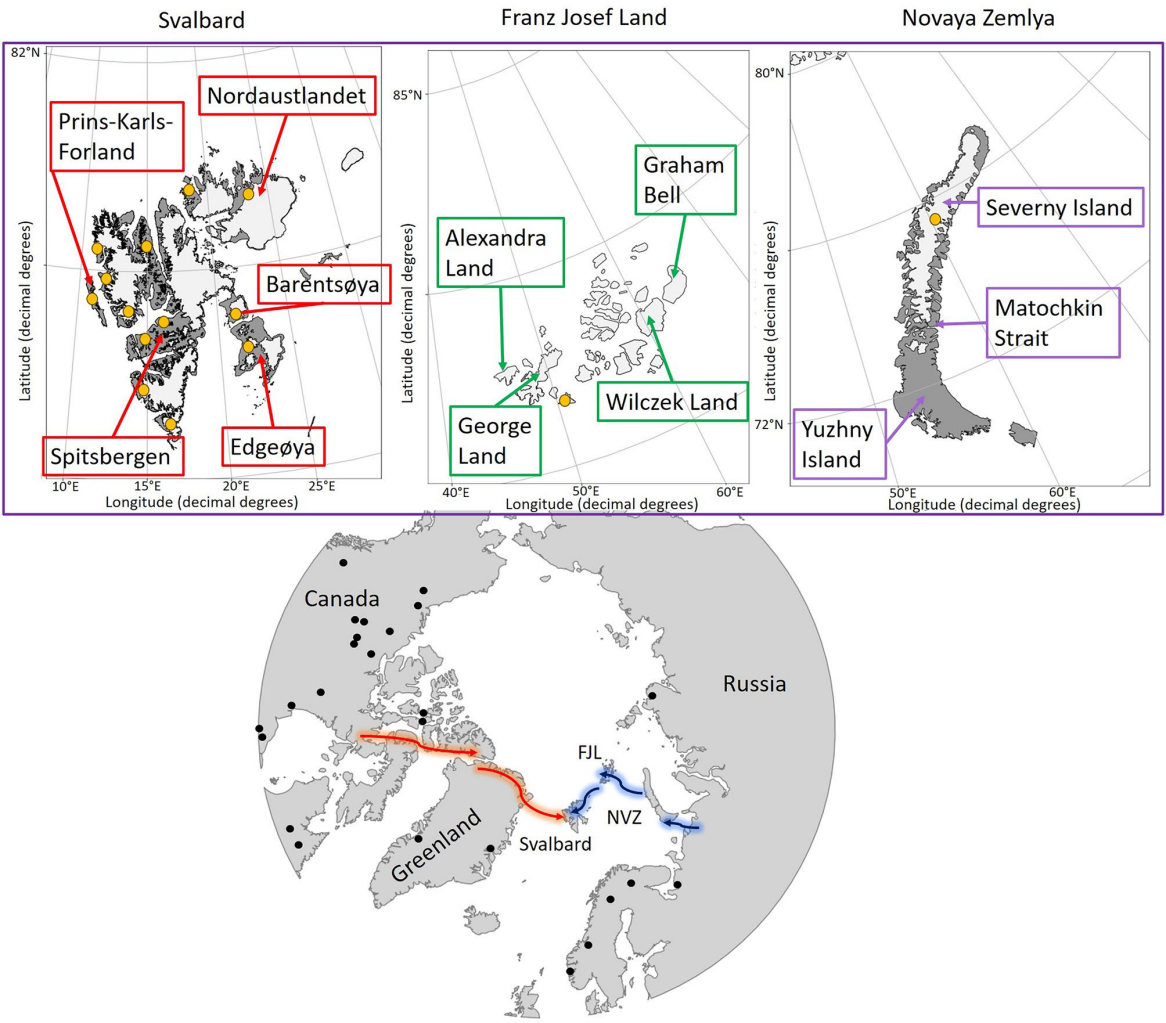
Map of the Holarctic illustrating the two main hypotheses of reindeer colonisation routes to Svalbard. The red arrows indicate the western route hypothesis, and the blue arrows indicate the eastern route hypothesis. Franz Josef Land is labelled as ‘FJL’ and Novaya Zemlya as ‘NVZ’. Additionally, the map includes close-ups of the three Eurasian Arctic archipelagos Novaya Zemlya, Franz Josef Land and Svalbard. Present-day glaciation is shown in the close-ups. Arrows point out the biggest islands or structures of each archipelago. Black and orange dots point out the approximate known sampling locations.

There is still uncertainty about the colonisation route taken by the Arctic island reindeer that now inhabit Svalbard (i.e., Svalbard reindeer, *R. t. platyrhynchus*). Two main hypotheses have been proposed. The first hypothesis proposes a North American origin (i.e., western route), stating that reindeer from the Arctic islands originate from a site in the Canadian Arctic archipelagos and spread eastward to Greenland and subsequently to Svalbard^29^ (Fig. 1). This hypothesis is supported by similarities in cranial measurements between individuals from Svalbard, specimens from Ellesmere Island and Axel Heiberg Island as well as extinct caribou from East Greenland (*R. t. eogroenlandicus*)^29^. Furthermore, previous studies identified a nuclear transferrin (TF) allele shared between Svalbard reindeer and Peary caribou (*R. t. pearyi*), as well as some other North American subspecies, which is entirely absent in Eurasian reindeer^30–32^.

The second hypothesis supports a Eurasian origin (i.e., eastern route). It states that the Svalbard population of *R. t. platyrhynchus* originates from the *R. t. tarandus* population of the Novaya Zemlya archipelago, possibly in a stepping-stone dispersal pattern via Franz Josef Land (Fig. 1)^4,33–36^. Previous phylogenetic^4,37^ and osteological^36^ studies support an eastern route based on shared haplotypes between Svalbard and Novaya Zemlya as well as the Taimyr peninsula. Congruent with these studies, the Svalbard rock ptarmigan (*Lagopus muta)* also shares a haplotype with its Taymyr populations, indicating an eastern route of colonisation and thus that other species may have used this dispersal route^11^. While there is partial support for both routes of colonisation in reindeer, the use of short mtDNA fragments in previous studies lacks resolution to properly infer the origin of Arctic island reindeer, and therefore uncertainty remains.

Colonisation of the new remote High Arctic habitat suggests that the species has a number of morphological, behavioural, physiological and metabolic adaptations, which are encoded by a number of genes^17,20,38^. Indeed, several nuclear genes (e.g. UCP-1; TRPA1) have been identified and associated with cold-adaptation in Arctic species^12,39^. As mitochondria play an essential role in cell processes such as energy production and thermoregulation, variation within the mitochondrial DNA (mtDNA) can also be involved in cold-adaptation^40^. Previous studies on mtDNA genes of other species indicate that there is a trade-off between energy or heat production and that the latter is favoured in cold-adapted species^40,41^.

Since reindeer/caribou have become extinct in parts of their former range, genetic diversity has likely been lost^21^. Therefore, modern samples alone may not be sufficient to disentangle the colonisation route to the High Arctic. Advances in ancient DNA (aDNA) extraction and sequencing now enable the use of the subfossil remains of organisms that lived thousands of years ago^42,43^. Hence, aDNA has proven to be a powerful tool to study genetic variation that existed in the past^42,43^. Examining ancient genomes thus allows the direct observation of recent evolutionary change, enabling a better understanding of changes in biodiversity and adaptation patterns^43,44^. However, aDNA sequences are sprone to contamination and DNA damage^45^. Since mtDNA is more abundant than nuclear DNA, it is of great utility for evolutionary studies, especially in cases where endogenous content is too low for sequencing the entire nuclear genome. Moreover, mtDNA evolves approximately 4-10 times faster than nuclear DNA, which enables the estimation of divergence times among very closely related lineages and within a species^46^. To date, the mitogenomes of many extinct temperate and Arctic species (e.g. quagga (*Equus quagga*), cave bear (*Ursus spelaeus*), mammoths (*Mammuthus sp*.), moose (*Alces alces*), wattlebirds (*Callaeidae*)) have provided a wealth of knowledge on Pleistocene evolutionary biology and ecology^47–51^.

Here, we sequenced complete mitogenomes from ancient (i.e. > 500 years BP), historical (i.e. ~ 70–500 years BP), and modern reindeer specimens and analysed them together with previously published whole mitogenomes representing most of the *Rangifer tarandus* Holarctic distribution. We reconstructed the species’ range-wide phylogeny and inferred the timing and route of colonisation of the Arctic archipelagos Svalbard, Franz Josef Land and Novaya Zemlya. Furthermore, we leveraged this large dataset and analysed mtDNA protein coding genes of Svalbard reindeer to identify possible signatures of positive selection associated with adaptation to the High Arctic environment.

## Results

### Mitogenome sequencing

To reconstruct the phylogeny of reindeer across the whole Holarctic region, investigate the origin and route of colonisation of the Arctic archipelagos by *Rangifer tarandus* and examine the mitogenomic basis of its adaptation to the arctic environment, we analysed 123 published and 51 newly-sequenced ancient and historical (i.e., 4752–100 years BP) as well as modern whole mitogenomes. The newly-sequenced ancient and historical samples (n = 31) had a mean sequencing depth ranging from 6× to 2131×, and an endogenous content ranging from 3 to 89% (Table S1). Some mitogenomes were excluded due to low quality (see “Methods”), thus a reduced mitochondrial alignment of 166 mitogenomes with a length of 16,349 bp was used to construct a haplotype network. Furthermore, a subset of 83 mitogenomes with a length of 16,351 bp was used to construct a tip-dated Bayesian phylogeny. The data showed that the extinct East Greenland caribou, Svalbard reindeer, and Novaya Zemlya reindeer have the lowest nucleotide diversity, at least an order of magnitude lower than in any other population previously analysed (Table 1).

**Table 1:** Overview of the nucleotide diversity, haplotype diversity, number of segregating sites and total number of haplotypes in the different geographic populations*.*

Population	Nucleotide diversity	Haplotype diversity	Number of segregating sites	Total number of haplotypes
Modern Canada (n = 24)	0.00597 (± 0.00031)	1.000 (± 0.012)	463	24
Modern Svalbard (n = 96)	0.00044 (± 0.00001)	0.874 (± 0.012)	36	15
Historical and ancient Svalbard (n = 17)	0.00038 (± 0.00006)	0.993 (± 0.023)	36	16
Modern Russia (n = 9)	0.00518 (± 0.00048)	1.000 (± 0.052)	123	9
Modern Novaya Zemlya (n = 5)	0	0	0	1
Modern Norway (n = 4)	0.00213 (± 0.00093)	0.833 (± 0.222)	68	3
Historical and ancient Norway (n = 1)	0	0	0	1
Historical and ancient Franz Josef Land (n = 3)	0.00293 (± 0.00128)	1.000 (± 0.272)	66	4
Modern Finland (n = 3)	0.00405 (± 0.00183)	1.000 (± 0.272)	98	3
Historical and ancient Sweden (n = 7)	0.00160 (± 0.00104)	0.524 (± 0.209)	41	3
Modern West Greenland (n = 2)	0.00037 (± 0.00018)	1.000 (± 0.500)	6	2
Historical and ancient East Greenland (n = 3)	0	0	0	1

Sample sizes are reported for the different sample groups as (n = X). The number following the ± stands for standard deviation.

### Haplotype network

**Figure 2:**
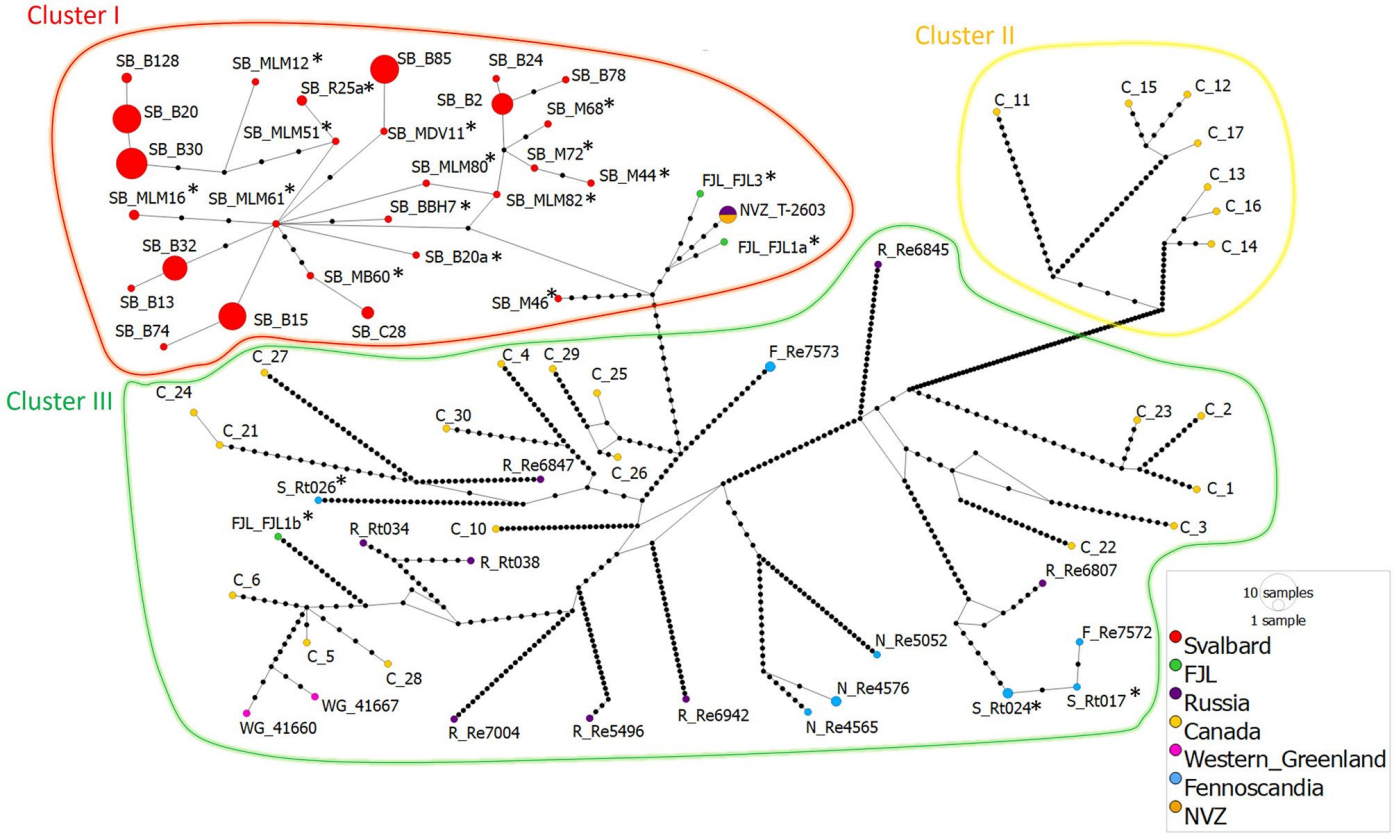
Median-joining haplotype network inferred from 166 whole mitogenome sequences of reindeer. The three main haplotype clusters (I–III) are circled in red, yellow and green, respectively. Cluster I and III = BEL and Cluster II = NAL. Each circle within the clusters represents a unique haplotype and its size reflects the number of individuals with that haplotype. Note that the branch length is not scaled to the number of substitutions and that the labels correspond to the first name of the individual with this haplotype appearing in the input file. Each small black circle indicates one nucleotide difference between haplotypes. Colour codes listed in the legend denote the geographic origin of the population. Fennoscandia combines the samples from Sweden, Finland and Norway. Geographic location abbreviation in sample names, SB: Svalbard; FJL: Franz Josef Land; R: Russia; NVZ: Novaya Zemlya; C: Canada; WG: Western Greenland; S: Sweden; N: Norway, F: Finland. Asterisks indicate ancient and historical samples.

The haplotype network analysis revealed three distinct clusters (i.e., I, II and III; Fig. 2). Among the 166 reindeer mitogenomes, a total of 73 distinct mitochondrial haplotypes were identified (Fig. 2). The highest haplotype diversity is found in Canada, which includes several lineages and ecotypes (Table S1).

Cluster I shows a star-like structure and comprises 27 haplotypes represented by 114 Svalbard individuals. Nested within this cluster are two of the three individuals from Franz Josef Land, each represented by its own unique haplotype, and the five individuals from Novaya Zemlya represented by a single haplotype. The haplotype from one ancient Svalbard sample (SB_MLM61; ca. 1875 years BP) is at the centre of cluster I. All other cluster I haplotypes stem from this one in a star-like pattern, indicating that it may be ancestral. Closest to the centre of the cluster I are mostly haplotypes found in single ancient individuals. Haplotypes found in modern individuals from Svalbard are located on cluster I’s outer branches. Two of the three Franz Josef Land haplotypes and the Novaya Zemlya haplotype group on an outer branch of cluster I with one of the two oldest ^14^C-dated samples (SB_MLM82; 3127 years BP), slightly outside of the centre of the network.

Cluster II is the most divergent among the three clusters, showing the highest number of substitutions (i.e. 64) between branches. It comprises seven individuals from Canada, each represented by its own unique haplotype, distributed in two smaller branches. Finally, cluster III comprises 32 haplotypes from most sampled populations (i.e., Canada, Finland, Russia, Sweden, Norway, Greenland and Franz Josef Land) across four smaller branches with no obvious geographical structuring.

### Bayesian phylogeny and timing of divergence

**Figure 3:**
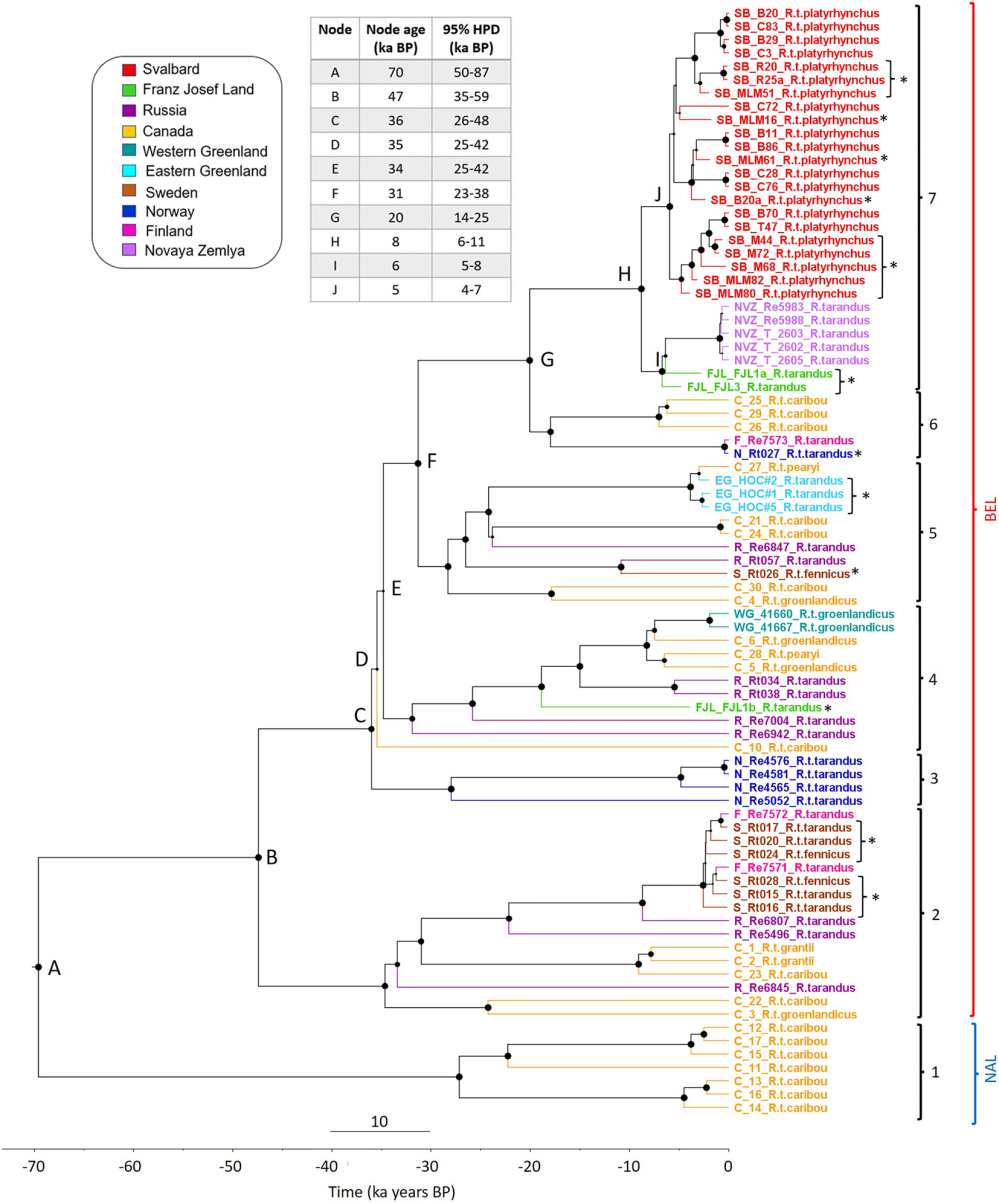
Bayesian phylogenetic tree based on 83 whole mitogenome sequences from modern, historical and ancient reindeer. To facilitate the visualisation of the phylogeny, the number of Svalbard sequences was randomly reduced to a subset of 22 mitogenomes (i.e.; 11 historical and ancient and 11 modern ones). Asterisks indicate historical and ancient samples. Clades 1–7 are indicated with curly brackets. Clade 1 exclusively includes the individuals of the NAL lineage as indicated by a blue curly bracket and clades 2–7 include the individuals of the BEL lineage as indicated by a red curly bracket. Samples are coloured by geographical location as listed in the legend and labels include the subspecies. Major nodes are labelled alphabetically and their divergence times are listed as mean node age and 95% highest posterior density (HPD) given in thousands of years (ka) in the upper left corner. Posterior probability (PP) node support over 0.72 is indicated by black node circles. PP of the major nodes is > 0.9 except for nodes D and E (0.36 and 0.35 respectively).

The Bayesian phylogeny revealed seven clades and 10 major nodes (A–H; Fig. 3) with strong statistical support (PP > 0.92) except for nodes D and E (PP = 0.35–0.36). The earliest branching clade 1 comprises individuals from Canada that belong to the NAL. There is little geographical structure within clades 2–6, and Eurasian and Canadian samples are distributed across the branches. All these clades belong to the BEL. In clade 7, all of the modern, historical and ancient Svalbard individuals form a group that is sister to a clade containing two of the three samples from Franz Josef Land and the five samples from Novaya Zemlya. Clade 3 comprises all four modern Norwegian individuals. The estimated mean ages for the historical samples from Eastern Greenland range from 1968 to 1870 years BP (Table S2) and have 95% highest posterior density (HPD) values ranging from 0 years BP to 4701 years BP.

The earliest split in the phylogenetic tree separates the NAL and the BEL at 69,577 years BP (95% HPD: 50,871–87,202 years BP; node A, Fig. 3). The most recent split between parts of the BEL and the Svalbard, Franz Josef Land and Novaya Zemlya archipelagos is estimated at 20,065 years BP (95% HPD: 14,487–25,666 years BP, node G), and the divergence between Svalbard and Franz Josef Land/Novaya Zemlya is estimated at 8792 years BP (95% HPD: 6526–11,256 years BP, node H). The split between the two Franz Josef Land samples is estimated at 6710 years BP (95% HPD: 5235–8362 years BP, node I), and the first divergence among Svalbard individuals at 5925 years BP (95% HPD: 4415–7585 years BP, node J). The estimated posterior substitution rate for the mitogenomes based on the inferred ^14^C dates of ancient samples and of historical and modern samples with a known year of death was 9.4148 × 10^–8^ substitutions/site/year (95% HPD Interval: 5.84 × 10^–8^, 1.31 × 10^–7^).

### Positive selection and adaptation to cold environments

We tested for evidence of positive selection in modern cold-adapted Svalbard reindeer using the random effects and Bayesian approaches implemented in MEME and FUBAR, respectively, which estimate the ratio of synonymous and non-synonymous mutations (i.e., ω = dN/dS) at a given site. Using MEME, we found no indication for positive selection in any mitochondrial genes. In contrast, FUBAR showed evidence for positive selection in only a single codon (i.e., position 205; AA Lysine) within gene ND4 of Complex I, with a posterior probability of 0.922 and an ω value of 7.325 (synonymous α = 4.950, non-synonymous β = 36.260).

## Discussion

Here, we used 174 ancient, historical and modern mitogenomes to resolve the timing, origin and route of colonisation of the Arctic islands by reindeer and to investigate the genetic basis of adaptation to the Svalbard environment. First, in agreement with previous studies, we identified two distinct mtDNA reindeer lineages: NAL and BEL^23–27^. Second, our dataset revealed Franz Josef Land and Novaya Zemlya as closest sister clades to Svalbard, thereby indicating a recent shared ancestry for these Arctic islands. Furthermore, we found some evidence for positive selection within the mtDNA of the cold-adapted Svalbard reindeer.

Consistent with Taylor et al.^27^ our phylogenetic tree identifies the same two mtDNA lineages. Seven Canadian samples that diverged ca. 70,000 years BP in our phylogeny most likely belong to the NAL, whereas all other samples belong to the BEL lineage. Furthermore, mitogenomes from different subspecies are found throughout the tree thus showing inconsistencies with the current reindeer taxonomy. Our estimate of the divergence time between the NAL and BEL lineages based on a tip-dated phylogeny of whole mitochondrial genomes (ca. 50,000–87,000 years BP) overlaps with the estimate of Klütsch et al.^23^ (i.e., 44,600–135,800 years BP) based on microsatellite data and mtDNA control region haplotypes, as well as that of Polfus et al.^24^ (i.e., 68,600–173,600 years BP). However, our estimate is older than that of McDevitt et al.^26^ (28,100–46,700 years BP) and in stark contrast with that of Yannic et al.^25^ (184,000–430,000 years BP). The inconsistency between our estimate and those in these two studies^25,26^ is likely due to the fact that they used control region and mtDNA cytochrome b sequences, respectively, whereas we have used whole mitogenome sequences as well as ancient radiocarbon dated samples. In comparison to fossil-based approaches^25^, which use the most recent common ancestor of the Odocoleini for calibration, tip-dating incorporates multiple radiocarbon-dated samples, thus improving accuracy of divergence times and preventing underestimation in cases where clades are older than their oldest known fossil^52,53^. Future approaches using a broader dataset of ancient genomes and dated specimens, especially North American individuals (NAL), may be able to provide more precise information on the timing of divergence of the two lineages.

Moreover, in accordance with the results of Taylor et al.^27^, two individuals from Western Greenland formed a sister group to one of the two Peary caribou individuals (C_28), thus supporting a close relationship between modern Peary caribou and Greenland caribou. In contrast, the second Peary caribou individual (C_27) forms a sister group to the three ancient and extinct Eastern Greenland caribou (*R. t. eogroenlandicus*) samples, possibly as a result of local adaptation as suggested by^27^. Furthermore, earlier studies found a close relationship between Peary caribou and the extinct Eastern Greenland caribou based on morphological evidence as well as shared haplotypes^37,54^. The three Eastern Greenlandic samples were not radiocarbon-dated but the mean age estimates based on the phylogeny range from 1968 to 1870 years BP, suggesting that they likely are part of the extinct subspecies. This is further supported since they do not branch with the Western Greenlandic samples, which have been identified as belonging to the extant Greenland caribou subspecies (*R. t. groenlandicus*). However, the molecular age ranges for the three samples from Eastern Greenland overlap with 0 years BP, suggesting a possibility of being modern and belonging to the extant Greenland caribou subspecies. To ultimately confirm the age of these samples, it would be necessary to radiocarbon date them. Nonetheless, simply relying on their phylogenetic placement, it seems likely that they are part of the extinct subspecies. The remaining unresolved clades 2–6 of the phylogeny show little geographic structure, most likely due to admixture or introgression between lineages^55^.

The dated phylogeny estimated the divergence time between most of Eurasia and the Arctic islands at ca. 20,000 years BP. This estimate coincides with the end of the LGM ca. 20,000–18,000 BP and the beginning of a warmer period that initiated glacial retreat^20,37^. During the Weichselian glaciation (ca. 115,000–12,000 BP) reindeer established large populations covering most of northern Eurasia. These populations gradually began to retreat northward at the end of the Holocene, reaching as far as the High Arctic archipelagos^20^. However, since the deglaciation of these Arctic islands began slightly later ca. 13,000–10,000 years BP, their environmental conditions at the end of the LGM were not yet suitable for reindeer due to insufficient vegetation cover^37,56–59^. The colonisation of the archipelagos therefore must have occurred later in the early Holocene (from 10,000 years BP onwards) when the climate became milder^37,57^. Receding glaciers, warmer temperatures and reduced snow-cover during the early- to mid-Holocene (9000–6000 years BP) facilitated growth of various plants such as mosses, dwarf shrubs, lichens and grasses on the High Arctic archipelagos, thus providing favourable and extended vegetation sources for reindeer^21,57–59^. Consistent with these studies on the past climate and vegetation of the archipelagos, our Bayesian approach estimates the divergence of reindeer of the Eurasian Arctic islands at ca. 8000 years BP. The earliest published evidence for reindeer on Svalbard is based on reindeer faeces that were found in peat deposits radiocarbon-dated to 3800–5000 years BP^59^ whereas ancient antlers were recently dated up to 7,080 years BP (M. Le Moullec, B. B. Hansen & M. Martin, unpubl. data,^60^). Furthermore, ancient antlers from Franz Josef Land were radiocarbon dated at ca. 6400 years BP^21^. Consistent with these dates, our phylogeny supports a divergence between the two ancient samples from Franz Josef Land at ca. 6700 years BP and a divergence between the ancient and modern Svalbard samples slightly more recently at ca. 5900 years BP. For Novaya Zemlya, there are no radiocarbon dated estimates available and our phylogeny only includes two modern individuals. However, given geomorphologic studies on the Eurasian ice-sheet and the close relationship to the other archipelagos revealed by genetic studies, a colonisation at that time would seem plausible^4,37,61^. The close relationship between the contemporary and extinct reindeer populations of Svalbard, Franz Josef Land and Novaya Zemlya are thus consistent with a scenario where Svalbard was colonised from the east.

Surprisingly, one sample from Franz Josef Land is placed in a different clade than the other ones. FJL1b is radiocarbon dated to 3896 years BP and clusters in clade 4 with two samples from Russia. However, this radiocarbon date cannot be further corroborated by damage patterns as the sample was USER-treated. A possible explanation for this clustering may be that FJL1b represents a secondary colonisation, since it is still unclear whether Franz Josef Land had a suitable habitat to sustain a continuous population over time or rather if multiple extinction-colonisation events occurred^21,61,62^. Therefore, despite clustering with Russian samples, FJL1b most likely comes from Franz Josef Land.

Overall, our results are concordant with a previous study^4^ that found a shared haplotype among the three High Arctic islands and the Russian mainland, likely supporting an eastern route of colonisation. Additional contemporary evidence of such a dispersal route includes an observation of a live reindeer in Svalbard in 1912 bearing antler markers by the people of Novaya Zemlya, possibly also indicating contact to the mainland and confirming that movement across the ice was indeed possible^33,35^.

In contrast to earlier studies based on transferrin variation, we did not find a close relationship between Peary caribou, other North American caribou and Svalbard reindeer^30–32^. A more recent study based on sequences of the mtDNA control region^6^ found a shared haplotype between Svalbard reindeer and caribou from Quebec, suggesting a North American colonisation route. However, this haplotype is the same one previously found on the Taimyr Peninsula^37^, thus equally supporting an eastern route of colonisation.

It is worth noting that the absence of ancient mitogenomes from Canada and Siberia in our dataset potentially limits our understanding of the colonisation history of the Arctic islands. For instance, our results show that the clade closest to that of the Eurasian Arctic islands reindeer comprises individuals from Canada and Fennoscandia rather than Russia, thus challenging a unidirectional eastern origin hypothesis. Furthermore, the dispersal history of caribou is complex, with strong evidence for admixture between the NAL and BEL lineages^23,24,27,28^. Nevertheless, evidence based on nuclear genomes^60,63^ shows a closer affinity between Svalbard and Russian reindeer and thus supports an eastern route of colonisation, consistent with the interpretation based on our mtDNA data. Moreover, in the considered time frame (since the end of the LGM, ca. 10–12 ka BP), a direct colonisation from east or west is a more likely hypothesis than circumpolar dispersal from Taimyr across Northern Siberia, Alaska, Canada and Greenland.

Colonisation of its new High Arctic habitat must have exposed reindeer to a number of selective pressures, due to different environmental and thermal conditions compared to the mainland^38^. To produce and retain heat, reindeer require a number of physiological adaptations for an enhanced thermoregulation. Mitochondria are responsible for energy and heat production and both processes rely on a proton gradient that is generated during the process of oxidative phosphorylation (OXPHOS)^41^. Because caloric intake needs to be allocated to either energy production (i.e. coupled OXPHOS) or heat generation (i.e. uncoupled OXPHOS), there is a trade-off between those processes^41,64^. In an arctic environment, mutations in the mtDNA selecting the generation of heat rather than energy can occur and seem likely^38,41,64,65^.

The FUBAR method found evidence for positive selection in the gene ND4 of Complex I in the OXPHOS pathway. Complex I is the first and largest of the five complexes that make up the OXPHOS pathway^64^. It is responsible for the provision of electrons enabling the translocation of protons across the inner mitochondrial membrane creating the pH gradient and the subunit ND4 acts as a direct proton pump^41,64^. Hence, a change in sequence in this gene could potentially be of adaptive value, especially in regards to environmental pressures such as cold-stress, hypoxia or nutrient availability^41^. While thermoregulation is a biological function likely under selection in Svalbard reindeer, there are other environmental factors such as nutrient availability that may affect the OXPHOS trade-off^41^. In contrast to cold-adaptation, starvation or a shortage of nutrients may act as a force to generate ATP more efficiently, resulting in the exact opposite effect of enhancing the coupling of energy production in the OXPHOS pathway^41^. Importantly, signatures of positive selection in the mtDNA involving gene ND4 and support for the trade-off hypothesis have been found in several species adapted to different climates and habitats (e.g., fish^41,66^, birds^67^, insects^65^, arachnids^40^). In light of the environmental conditions of reindeer, it seems possible that the site under positive selection induces an elevated efficiency of OXPHOS uncoupling, resulting in an enhanced heat production. In addition to uncoupling as a mechanism for cold-adaptation, mitochondrial proteins may also impact the production of energy and heat through changes in mtDNA copy number, transcription and translation, messenger RNA stability as well as protein stability^38,40^. Camus et al.^65^ found that a sequence variation within the mtDNA, such as the present mutation on the positively selected site, may affect the copy number of mitogenomes and gene expression, therefore possibly enabling an adaptation to a cold climate. Further research on gene expression and the determination of mtDNA copy numbers are necessary to prove or rule out a possible impact on energy production through these factors.

In contrast to FUBAR^68^, there was no evidence for positive selection using MEME even though it is considered to be more sensitive because it considers both pervasive and episodic selection^69^. While MEME^70^ should show results that are consistent with methods only able to detect pervasive selection such as FUBAR, it is generally more powerful in detecting episodic selection. FUBAR is instead considered more robust than random effect models such as MEME, as it uses a more flexible and less restrictive setting and is thus less sensitive to model specifications^66^. It should be noted that it might be beneficial to study the nuclear genome in context of the uncoupled OXPHOS pathway and cold-adaptation, since Complex I also harbours subunits that are encoded by the nuclear genome and studies suggest that, despite the mtDNA evolving roughly 4-10 times faster, a coevolution of mtDNA and nuclear DNA is to be expected^71^.

Using aDNA, we show how species colonise remote islands in the Arctic and adapt to the environmental conditions of Svalbard. Furthermore, we refine previous divergence dates connected to glacial refugia and range shifts. Whole ancient genomes of reindeer as well as other terrestrial species would be essential to examine in more detail the history of colonisation of the Arctic archipelagos. Finally, additional data from Novaya Zemlya and Franz Josef Land would help to study patterns of adaptation to the conditions similar to Svalbard and to better resolve evolutionary relationships among the three islands.

## Methods

### Sample acquisition

The 31 historical and ancient samples (i.e., antlers, subfossil bones and dental calculus) were collected during several field trips on various sites of Svalbard (n = 17), Eastern Greenland (n = 3), Franz Josef Land (n = 3), Sweden (n = 7) and Norway (n = 1). Raw sequences for most ancient Svalbard samples (n = 16) were previously published by Kellner et al.^72^ and are available under the BioProject Accession no. PRJEB60484. ^14^C dates were calibrated using the IntCal20 calibration curve^73^. The whole mitogenomes from modern individuals comprise previously published samples from Svalbard (n = 96; ENA BioProject PRJEB57293 and PRJEB61721) and Russia (n = 1; ENA BioProject PRJEB57293 and PRJEB61721) as well as newly sequenced genomes from Northern Finland (n = 3), Norway (n = 4) and several locations in Russia (n = 13). Raw sequences for the modern Canadian samples (n = 24) and the modern Western Greenland samples (n = 2) were downloaded from GenBank (NCBI) under the BioProject Accession No. PRJNA634908^27^.

### DNA extraction and sequencing

Historical and ancient samples were processed using various protocols in dedicated, positively-pressurised ancient DNA laboratory facilities at the Centre for Palaeogenetics (Stockholm), the NTNU University Museum, and Uppsala University. All procedures were performed using sterilised equipment and working surfaces to minimise the risk of exogenous and cross-sample contamination. For the three samples from Eastern Greenland (HOC#1,2,5) and one sample from Franz Josef Land (FJL1b), DNA was extracted from ca. 50 mg of bone or antler powder. To prevent exogenous contamination of the bone powder, bones were wrapped in aluminium foil except for the small drilling area and the drilling bit was changed after removing the outer part of the bone. DNA extraction was carried out following the control protocol described in Dehasque et al.^44^ and included two negative controls (extraction blanks), and double-stranded DNA (dsDNA) libraries were built following the protocol of Meyer and Kircher^74^, including two additional negative controls (library blanks) per 10 samples, with an additional incubation step removing deaminated cytosine sites (considered damage patterns) through uracil-DNA-glycosylase and endonuclease VIII (USER) treatment^75^. Lastly, libraries were sequenced on either 2 × 50 bp chemistry on an Illumina SPrime or 2 × 100 bp chemistry on the Illumina NovaSeq S4 at the National Genomics Infrastructure (NGI, Stockholm, Sweden). Four extraction blanks processed alongside the first 20 reindeer samples extracted with this method were sequenced and ran through a metagenomics pipeline, which showed that they contained< 0.01% reads assigned to mammals, indicating no reindeer cross contamination into the blanks.

The seven historical samples from Sweden, one historical sample from Norway and three modern samples from Russia (Table S1) were collected as dental calculus scraped from the individuals’ teeth. Surface decontamination consisted of 10 min of UV light exposure and 30 s of EDTA wash, as has been previously described in Brealey et al.^76^. Next, DNA was extracted from ca. 5–20 mg of dental calculus following a silica-column based extraction method^77^. An extraction blank was carried out alongside the samples for every extraction batch. dsDNA libraries for calculus samples, extraction blanks and library blanks (no-template controls) were prepared following van der Valk et al.^78^ without USER treatment. Libraries were sequenced using an Illumina HiSeq 2500 platform and using 2 × 150 bp chemistry and an additional two lanes on the same platform using 2 × 100 bp chemistry (SciLifeLab, Uppsala, Sweden).

For the ancient samples B16, B20a, M72, MLM12, MLM16, MLM51, MLM80, and MLM82 from Svalbard, ca. 50 mg of bone/antler powder was subjected to a DNA extraction protocol based on a previous study^79^. Briefly, bone powder was agitated for 48 h at 55 °C in 1 mL lysis buffer^80^ consisting of 0.1 M urea, 0.45 M EDTA, 0.15 mg/mL proteinase K, and molecular biology H_2_O. 10 µL of 20 mg/mL proteinase K was added prior to the final 20 h of lysis. An extraction blank was carried out alongside the samples for every extraction batch. Then the protocol of Dabney and Meyer^79^ was used with bleach-sterilised Zymo-Spin V column extenders. Dual-indexed dsDNA libraries were constructed on the eluted DNA, extraction blanks and a single library blank (no-template control) following the protocol of Meyer and Kircher^74^ without USER treatment. These libraries were subjected to target enrichment with *Rangifer tarandus* mitochondrial DNA baits provided by Arbor Biosciences (Product Code 303048) using the MYbaits protocol v.3.02. In-solution hybridization was performed at 55 °C for 28 h. Thereafter, the pre- and post-capture libraries were sequenced on an Illumina MiniSeq instrument with 1 × 75 bp or 2 × 75 bp chemistry (NTNU University Museum) and an Illumina NextSeq 550 instrument with 1 × 75 bp chemistry (NTNU Genomics Core Facility).

All other historical and ancient samples were extracted from ca. 48–360 mg of bone/antler powder which was drilled after surface decontamination by wiping with bleach, using a silica-pellet based extraction protocol based on Rohland and Hofreiter^81^. Briefly, a digestion buffer consisting of 1.25% (v/v) proteinase K (20 mg/mL), 90% (v/v) EDTA (0.5 M) and 8.75% (v/v) molecular-grade water was used for digestion. Samples were pre-digested in 1 mL digestion buffer for 10 min at 37 °C on a rotor. The samples were spun down, the pre-digest was removed, and 4 mL digestion buffer was added to the samples. Samples were left for digestion on a rotor for 18 h at 37 °C. The lysis buffer was mixed 1:10 with Qiagen PB buffer modified by adding 9 mL sodium acetate (5 M) and 2 mL NaCl (5 M) to 500 mL of Qiagen PB buffer. pH was adjusted to 4.0 using concentrated (37%/12 M) HCl. 50 µL in-solution silica beads were added, and samples were left on a rotor for 1 h at ambient temperature to allow binding of the DNA. Silica pellets with bound DNA were purified using Qiagen MinElute purification kit following the manufacturer's instructions and eluted in 65 µL Qiagen EB buffer. An extraction blank was carried out alongside the samples for every extraction batch. Then dual-indexed dsDNA libraries were constructed on the eluted DNA samples, extraction blanks, and a single library blank (no-template control) following the BEST v.1.1 protocol^82^. Thereafter, libraries were sequenced on an Illumina MiniSeq (NTNU University Museum), an Illumina NextSeq 550 (NTNU Genomics Core Facility), or an Illumina NovaSeq 6000 platform using 2 × 150 bp chemistry (Norwegian National Sequencing Centre, Novogene UK).

For the modern samples, DNA was extracted from ca. 20 mg of skin or muscle tissue alongside an extraction blank using a DNeasy Blood and Tissue Kit (Qiagen, Hilden, Germany) following the manufacturer’s instructions. For bone and antler samples minor modifications were applied as previously described in Peeters et al.^83^. Libraries were then either prepared in-house or at Novogene UK. For in-house library preparation, DNA was subsequently sheared to a fragment length of 400 bp using a Covaris ME220 focused ultrasonicator. Next, libraries were prepared from the sheared DNA using a blunt-end, single-tube protocol for double-stranded DNA (BEST v.1.1)^82^. For libraries prepared at Novogene UK, DNA was sheared to a fragment length of 350 bp and subsequently end-repaired, A-tailed and ligated with Illumina adapters. Libraries were then either sequenced on an Illumina HiSeq 4000 platform using a 2 × 150 bp set up at the NTNU Genomics Core Facility or on an Illumina NovaSeq 6000 platform using 2 × 150 bp set up at Novogene, UK.

### Bioinformatic procedures

Raw sequence reads of the ancient samples were trimmed of adapters and internal barcodes, reads shorter than a length of 30 bp were discarded and paired-end reads were merged using fastp v.0.23.2^84^. Subsequently, the merged reads were mapped to a reference mitogenome of *Rangifer tarandus* (GenBank Accession: NC_007703.1) using the BWA aln algorithm v.0.7.17^85^.

Next, the alignments were converted from SAM to BAM format and coordinate sorted and indexed using SAMtools v.1.9^86^. PCR duplicates were then removed using the samremovedup.py python script (https://github.com/pontussk/samremovedup). For samples that were sequenced with multiple runs, reads were merged using SAMtools merge v.1.9.

Mapped reads were then imported into Geneious Prime v.2022.2.2 (https://www.geneious.com)^87^ and consensus sequences were called for each sample using the majority call rule with a minimum depth of 5 × per position. Any ambiguous positions remaining as well as positions with no coverage at all were called as “N”. Variants and sites where two bases remained in a 50:50 ratio, were visually inspected and called as “N” in the consensus sequences to avoid incorporation of erroneous SNPs.

Endogenous DNA content (defined as the number of reads mapping to the reference mitogenome divided by the total number of reads prior to duplicate removal) for the historical and ancient samples was estimated from the BAM files before duplicate removal using SAMtools flagstat v.1.9. The sequences were then aligned using the MUSCLE v.3.8.425^88^ plug-in in Geneious Prime v.2022.2.2 with a maximum number of iterations of 8. Afterwards, remaining indels in the alignment were visually detected and removed.

Modern samples were processed using the GenErode bioinformatics pipeline^89^. Briefly, raw sequences were trimmed of adapters using fastp v.0.22.0 and then mapped to the *Rangifer tarandus* mitogenome reference using the BWA mem algorithm v.0.7.17. Next, reads were sorted and PCR duplicates were removed using SAMtools v.1.12. The resulting BAM files were then subsampled to an average depth of 93–100 × using SAMtools view v.1.9 and subsequently imported into Geneious Prime v.2022.2.2, where consensus sequences were called as described above.

In this study we generated and collated data for a total of 174 mitogenomes. Within this dataset, we constructed a haplotype network based on 166 of these mitogenomes whereas a subset of 83 mitogenomes was used for the construction of a phylogenetic tree (see details below).

### Beast phylogeny and haplotype network

To quantify the haplotype diversity and examine the relationships among populations and islands, we built a haplotype network for 166 of the total 174 mitogenomes from all over the Holarctic range. The alignment was then exported in nexus format with a traits block containing a division into the nine sampling regions into PopART v.1.7^90^. Since PopART masks any columns in the alignment with ambiguous or missing sites (e.g., ‘N’), several samples (n = 8; four historical Swedish, three ancient Eastern Greenland and one historical Russian) with a high proportion (i.e., ${\overline{\text{x}}}$ = 27.8%) of such sites were removed to avoid underestimation of haplotype number and an overall loss of resolution. The final dataset was then used to build a haplotype network using the median-joining option in PopART^90,91^.

Secondly, we built a phylogeny for modern, historical and ancient samples using BEAST v.1.10.4^92^. To facilitate the visualisation of the phylogeny, the number of Svalbard sequences was randomly reduced to a subset of 22 mitogenomes (i.e.; 11 historical and ancient and 11 modern ones), leading to a total of 83 mitogenomes used for the phylogeny. To estimate the molecular age of the undated samples, an ‘undated’ taxa partition was created using the ‘sampling with individual priors’ option. The median probability values of the ^14^C dated samples (cal BP) were used for the calibrated tip dates. Additionally, ten modern and historical samples from animals put-down either in 1994 or 1910 were also used as tip dates. The evolutionary model used for the mitogenome dataset was determined in jmodeltest2 v.2.1.9^93,94^ to be HKY with gamma distribution and invariant sites. A constant size model with a strict molecular clock and a clock rate with a uniform distribution and a mean value of 9.4148 × 10^–8^ substitutions/site/year (95% HPD Interval: 5.8434 × 10^–8^, 1.3088 × 10^–7^) was used for the construction of the phylogeny. The substitution rate was estimated in a preliminary run, using only the modern samples and the dated samples with their median ^14^C ages as tip dates. The ages of the undated samples were estimated using a uniform prior ranging from 0 to 15,000 years BP. Three independent trees were then run for 100 million generations with sampling every 1000 generations. All BEAST runs were checked visually for convergence (i.e., aiming for a minimum effective sample size for parameters above 200) in Tracer v.1.7.2^95^. Subsequently, the three individual trees and log files were combined after removing 10% burn-in (10 million generations) of each file using LogCombiner v.1.10.4. Next, TreeAnnotator v.1.10.4 was used to summarise the trees from the combined trees file into a single target tree. Lastly, the Bayesian tree was visualised and built in FigTree v.1.4.4^96^. Summary statistics on nucleotide diversity (π), haplotype diversity (Hd) and total number of segregating sites were calculated using DnaSP6 v.6.12.03^97^.

### Tests of positive selection

To test for evidence of positive selection in Svalbard reindeer, the reference and annotation for the *Rangifer tarandus* mitochondrial reference sequence (GenBank Accession: NC_007703.1) were downloaded and imported into Geneious. The annotated reference was then aligned to the modern Svalbard sample sequences (n = 96) using the MUSCLE v.3.8.425 plug-in in Geneious with a maximum number of iterations of 8. Next, non-coding regions and regions coding for tRNA and rRNA were removed manually.

To identify stop codons and remove the three corresponding nucleotides without causing frameshifts, the consensus nucleotide sequence was translated to an amino acid (AA) sequence using the translation tool for vertebrate mitochondrial DNA in Geneious. Due to the overlap in the annotation, protein coding regions of the mitogenomes were then separated into three different alignments. A first alignment ‘Reindeer 1’ containing genes ND1, ND2, COX1, COX2, ATP8, COX3, ND3, ND4L, ND5 and CYTB; a second alignment ‘Reindeer 2’ containing genes ATP6 and ND4 and a third alignment ‘Reindeer 3’ containing gene ND6. Finally, the alignment files were exported from Geneious in fasta format for further downstream analyses.

We identified synonymous and non-synonymous substitutions using the Mixed Effects Model of Evolution (MEME) method^70^ and the Fast Unconstrained Bayesian AppRoximation (FUBAR) method^67^ available at datamonkey.org^98,99^. For each method the alignment files were uploaded on datamonkey.org and the genetic code was set to vertebrate mitochondrial DNA code. The p-value threshold for MEME was set to 0.05 and the cut-off for the posterior probability for FUBAR was set to 0.9. The resulting FUBAR values for dS and dN were used to manually calculate ω. If ω = 1 the site is considered to evolve without selective pressure (i.e., neutrally), if ω < 1 the site is considered to be under negative selection (i.e., purifying selection) and if ω > 1 the site is under positive selection^100–102^.

### Maps

All maps included in this study were created using R v.4.42 (https://www.R-project.org/)^103^ in RStudio v2022.12.0.353 (http://www.rstudio.com/)^104^. The circular maps of the Holarctic were created using the ‘maps’ package (https://www.rdocumentation.org/packages/maps/versions/3.4.1) and the close-up maps of the archipelagos using the ‘ggplot2’ and ‘ggOceanMaps’ packages (https://mikkovihtakari.github.io/ggOceanMaps/)^105^.

### Supplementary Information

Supplementary Figures.

Supplementary Tables.

## Data Availability

The multiple sequence alignments used for the phylogenetic and haplotype network analyses have been deposited on the Dryad repository (https://doi.org/10.5061/dryad.tx95x6b53).

## References

[1] Hewitt G (2000). The genetic legacy of the Quaternary ice ages. Nature.

[2] Hofreiter M, Stewart S (2009). Ecological change, range fluctuations and population dynamics during the pleistocene. Curr. Biol..

[3] Avise JC, Walker DE (1998). Pleistocene phylogeographic effects on avian populations and the speciation process. Proc. R. Soc. Lond. B..

[4] Kvie KS (2016). Colonizing the high arctic: Mitochondrial DNA reveals common origin of Eurasian archipelagic reindeer (*Rangifer tarandus*). PLoS ONE.

[5] Stewart JR, Lister AM, Barnes I, Dalén L (2010). Refugia revisited: individualistic responses of species in space and time. Proc. R. Soc. B..

[6] Flagstad Ø, Røed KH (2003). Refugial origins of reindeer (*Rangifer tarandus* L.) inferred from mitochondrial DNA sequences. Evolution.

[7] Taberlet P, Fumagalli L, Wust-Saucy A-G, Cosson J-F (1998). Comparative phylogeography and postglacial colonization routes in Europe. Mol. Ecol..

[8] Pielou EC (1991). After the Ice Age: The Return of Life to Glaciated North America.

[9] Shafer AB, Cullingham CI, Côté SD, Coltman DW (2010). Of glaciers and refugia: A decade of study sheds light on the phylogeography of northwestern North America. Mol. Ecol..

[10] Dalén L (2005). Population history and genetic structure of a circumpolar species: The arctic fox. Biol. J. Linn. Soc..

[11] Sahlman T, Segelbacher G, Höglund J (2009). Islands in the ice: colonisation routes for rock ptarmigan to the Svalbard archipelago. Ecography.

[12] Lord E (2022). Population dynamics and demographic history of Eurasian collared lemmings. BMC Ecol. Evo..

[13] Landvik JY (2003). Northwest Svalbard during the last glaciation: Ice-free areas existed. Geology.

[14] Brochmann C (2004). Polyploid in arctic plants. Biol. J. Linn. Soc..

[15] Johansen BE, Karlsen SR, Tømmervik H (2012). Vegetation mapping of Svalbard utilising Landsat TM/ETM+ data. Polar Rec..

[16] Banfield, A. W. F. *A Revision of the Reindeer and Caribou, Genus Rangifer* (National Museum of Canada, Bulletin No. 177, 1961).

[17] Bevanger K, Jordhøy P (2004). Reindeer: The Mountain Noma.

[18] Harding LE (2022). Available names for Rangifer (Mammalia, Artiodactyla, Cervidae) species and subspecies. ZooKeys.

[19] IUCN (International Union for the Conservation of Nature). 2022. The IUCN red list of threatened species. https://www.iucnredlist.org. Viewed 26 July 2023.

[20] Holand Ø, Mizin I, Weladji RB, Corlatti L, Zachos FE (2022). Reindeer *Rangifer tarandus* (Linnaeus, 1758). Terrestial Cetartiodactyla. Handbook of the Mammals or Europe.

[21] Forman SL, Lubinski DJ, Weihe RR (2000). The Holocene occurence of reindeer on Franz Josef Land, Russia. The Holocene.

[22] Festa-Bianchet M, Ray JC, Boutin S, Côté SD, Gunn A (2011). Conservation of caribou (*Rangifer tarandus*) in Canada: An uncertain future. Can. J. Zool..

[23] Klütsch CFC, Manseau M, Trim V, Polfus J, Wilson PJ (2016). The eastern migratory caribou: The role of introgression in ecotype evolution. R. Soc. Open Sci..

[24] Polfus JL, Manseau M, Klütsch CFC, Simmons D, Wilson PJ (2016). Ancient diversification in glacial refugia leads to intraspecific diversity in a Holarctic mammal. J. Biogeogr..

[25] Yannic G (2014). Genetic diversity in caribou linked to past and future climate change. Nat. Clim. Change.

[26] McDevitt AD (2009). Survival in the Rockies of an endangered hybrid swarm from diverged caribou (*Rangifer tarandus*) lineages. Mol. Ecol..

[27] Taylor RS (2020). The role of introgression and ecotypic parallelism in delineating intraspecific conservation units. Mol. Ecol..

[28] Weckworth BV, Musiani M, McDevitt AD, Hebblewhite M, Mariani S (2012). Reconstruction of caribou evolutionary history in Western North America and its implications for conservation. Mol. Ecol..

[29] Hakala AVK, Staaland H, Pulliainen E, Røed KH (1986). Taxonomy and history of arctic island reindeer with special reference to Svalbard reindeer: A preliminary report. Rangifer.

[30] Røed KH, Witten KR (1986). Transferrin variation and evolution of Alaska reindeer and caribou, *Rangifer tarandus* L. [special issue]. Rangifer.

[31] Røed KH, Staaland H, Broughton E, Thomas DC (1986). Transferrin variation in caribou (*Rangifer tarandus* L.) on the Canadian Arctic islands. Can. J. Zool..

[32] Røed KH, Ferguson MA, Crête M, Bergerud TA (1991). Genetic variation in transferrin as a predictor for differentiation and evolution of caribou from eastern Canada. Rangifer.

[33] Hoel A (1916). Hvorfra er Spitsbergenrenen kommet?. Naturen.

[34] Nøis, D. N. Villreinen på Svalbard. *Polarboken,* 45–57 (1958).

[35] Lønø O (1959). Reinen på Svalbard. Naturen.

[36] Willemsen GF (1983). Osteological measurements and some remarks on the evolution of the Svalbard reindeer, *Rangifer tarandus platyrhynchus*. Z. Säugetierkd..

[37] Gravlund P, Meldgaard M, Pääbo S, Arctander P (1998). Polyphyletic origin of the small-bodied, high-arctic subspecies of tundra reindeer (*Rangifer tarandus*). Mol. Phylogenet. Evol..

[38] Das J (2006). The role of mitochondrial respiration in physiological and evolutionary adaptation. Bioessays.

[39] Lynch VJ (2015). Elephantid genomes reveal the molecular bases of wooly mammoth adaptations to the arctic. Cell Rep..

[40] Sun J-T (2018). Evolutionary divergence of mitochondrial genomes in two Tetranychus species distributed across different climates. Insect Mol. Biol..

[41] Sebastian W (2020). Signals of selection in the mitogenome provide insights into adaptation mechanisms in heterogeneous habitats in a widely distributed pelagic fish. Sci. Rep..

[42] Leonardi M (2017). Evolutionary patterns and processes: Lessons from ancient DNA. Syst. Biol..

[43] Bieker VC, Martin MD (2018). Implications and future prospects for evolutionary analyses of DNA in historical herbarium collections. Bot. Lett..

[44] Dehasque M (2022). Development and optimization of a silica column-based extraction protocol for ancient DNA. Genes.

[45] Gilbert MTP, Willerslev E (2006). Authenticity in ancient DNA studies. Med. Secoli..

[46] DeSalle R, Schierwater B, Hadrys H (2017). MtDNA: The small workhorse of evolutionary studies. Front. Biosci. (Landmark Ed).

[47] Higuchi R, Bowman B, Freiberger M, Ryder OA, Wilson AC (1984). DNA sequences from the quagga, an extinct member of the horse family. Nature.

[48] Stiller M (2010). Withering away: 25,000 years of genetic decline preceded cave bear extinction. Mol. Biol. Evol..

[49] Van der Valk T (2021). Million-year-old DNA sheds light on the genomic history of mammoths. Nature.

[50] Lubbe P (2022). Mitogenomes resolve the phylogeography and divergence times within the endemic New Zealand Callaeidae (Aves: Passerida). Zool. J. Linn. Soc..

[51] Meiri M, Lister A, Kosintsev P, Zazula G, Barnes I (2020). Population dynamics and range shifts of moose (*Alces alces*) during the late quaternary. J. Biogeogr..

[52] Arcila D, Pyron RA, Tyler JC, Ortí G, Betancur-R R (2015). An evaluation of fossil-tip dating versus node-age calibrations in tetraodontiform fishes (Teleostei: Percomorphaceae). Mol. Phylogenet. Evol..

[53] Heads M (2012). Bayesian transmogrification of clade divergence dates: A critique. J. Biogeogr..

[54] Roby DD, Thing H, Brink KL (1984). History, status and taxonomic identity of caribou (*Rangifer tarandus*) in Northwest Greenland. Arctic.

[55] Von Holdt BM, Brzeski KE, Wilcove DS, Rutledge LY (2018). Redefining the role of admixture and genomics in species conservation. Conserv. Lett..

[56] Forman SL (2004). A review of postglacial emergence on Svalbard, Franz Josef Land and Novaya Zemlya, northern Eurasia. Quat. Sci. Rev..

[57] Birks HH (1994). Late Weichselian environmental change in Norway, including Svalbard. J. Quat. Sci..

[58] Birks HH (1991). Holocene vegetational history and climatic change in west Spitsbergen-plant macrofossils from Skardtjørna, an arctic lake. The Holocene.

[59] Van der Knaap WO (1989). Past Vegetation and reindeer on Edgeoya (Spitsbergen) between c. 7900 and c. 3800 BP, studied by means of peat layers and reindeer faecal pellets. J. Biogeogr..

[60] Dussex N (2023). Adaptation to the High-Arctic island environment despite long-term reduced genetic variation in Svalbard reindeer. iScience.

[61] Siegert MJ, Dowdeswell JA (2004). Numerical reconstructions of the Eurasian Ice Sheet and climate during the late Weichselian. Quat. Sci. Rev..

[62] Zale R, Glazovskiy A, Näslund J-O (1994). Radiocarbon dating the extinct caribou on Franz Josef Land. Boreas.

[63] Torres-Oliva, M. *et al.* Whole-genome sequencing of reindeer (*Rangifer tarandus*) populations reveals independent origins of dwarf ecotypes and potential molecular mechanisms underpinning cold adaptation. Preprint at https://www.researchsquare.com/article/rs-3619721/v1 (2023)

[64] Fernández-Gutiérrez J (2020). Key role of quinone in the mechanism of respiratory complex I. Nat. Commun..

[65] Camus MF, Wolf JBW, Morrow EH, Dowling DK (2015). Single nucleotides in the mtDNA sequence modify mitochondrial molecular function and are associated with sex-specific effects on fertility and ageing. Curr. Biol..

[66] Consuegra S, John E, Verspoor E, Garcia de Leaniz C (2015). Patterns of natural selection acting on the mitochondrial genome of a locally adapted fish species. Genet. Sel. Evol..

[67] Morales HE, Pavlova A, Joseph L, Sunnucks P (2015). Positive and purifying selection in mitochondrial genomes of a bird with mitonuclear discordance. Mol. Ecol..

[68] Murrell B (2013). FUBAR: A fast, unconstrained Bayesian AppRoximation for inferring selection. Mol. Biol. Evol..

[69] Spielman SJ, Anisimova M (2019). Evolution of viral genomes: Interplay between selection, recombination, and other forces. Evolutionary Genomics Methods in Molecular Biology.

[70] Murrell B (2012). Detecting individual sites subject to episodic diversifying selection. PLoS Genet..

[71] Gershoni M (2010). Coevolution predicts direct interactions between mtDNA-encoded and nDNA-encoded subunits of oxidative phosphorylation complex I. J. Mol. Biol..

[72] Kellner, F. L. *et al.* A paleogenomic investigation of overharvest implications in an endemic wild reindeer subspecies. Preprint at 10.1101/2023.09.21.558762v1 (2023). 10.1111/mec.17274 38279681

[73] Crema ER, Bevan A (2021). Inference from large sets of radiocarbon dates: Software and methods. Radiocarbon.

[74] Meyer M, Kircher M (2010). Illumina sequencing library preparation for highly multiplexed target capture and sequencing. Cold Spring Harb. Protoc..

[75] Rohland N, Eadaoin H, Swapan M, Nordenfelt S, Reich D (2015). Partial uracil-DNA-glycosylase treatment for screening of ancient DNA. Philos. Trans. R. Soc. B..

[76] Brealey JC, Leitao HG, Hofstede T, Kalthoff DC, Guschanski K (2021). The oral microbiota of wild bears in Sweden reflects the history of antibiotic use by humans. Curr. Biol..

[77] Dabney J (2013). Complete mitochondrial genome sequence of a Middle Pleistocene cave bear reconstructed from ultrashort DNA fragments. Proc. Natl. Acad. Sci. USA.

[78] Van der Valk T, Vezzi F, Ormestad M, Dalén L, Guschanski K (2020). Index hopping on the Illumina HiseqX platform and its consequences for ancient DNA studies. Mol. Ecol. Resour..

[79] Dabney J, Meyer M (1963). Extraction of highly degraded DNA from ancient bones and teeth. Methods Mol. Biol..

[80] Brace S (2012). Serial population extinctions in a small mammal indicate Late Pleistocene ecosystem instability. Proc. Natl. Acad. Sci. USA.

[81] Rohland N, Hofreiter M (2007). Ancient DNA extraction from bones and teeth. Nat. Protoc..

[82] Carøe C (2018). Single-tube library preparation for degraded DNA. Methods Ecol. Evol..

[83] Peeters B (2020). Sea ice loss increases genetic isolation in a high Arctic ungulate metapopulation. Glob. Change Biol..

[84] Chen S, Zhou Y, Chen Y, Gu J (2018). fastp: An ultra-fast-all-in-one FASTQ preprocessor. Bioinformatics.

[85] Li H, Durbin R (2009). Fast and accurate short read alignment with Burrows–Wheeler transform. Bioinformatics.

[86] Li H (2009). The sequence alignment/map format and SAMtools. Bioinformatics.

[87] Kearse M (2012). Geneious basic: An integrated and extendable desktop software platform for the organization and analysis of sequence data. Bioinformatics.

[88] Edgar RC (2004). MUSCLE: A multiple sequence alignment method with reduced time and space complexity. BMC Bioinform..

[89] Kutschera VE (2022). GenErode: A bioinformatics pipeline to investigate genome erosion in endangered and extinct species. BMC Bioinform..

[90] Leigh JW, Bryant D (2015). PopART: Full-feature software for haplotype network construction. Methods Ecol. Evol..

[91] Bandelt H, Forster P, Röhl A (1999). Median-joining networks for inferring intraspecific phylogenies. Mol. Biol. Evol..

[92] Suchard MA (2018). Bayesian phylogenetic and phylodynamic data integration using BEAST 1.10. Virus Evol..

[93] Darriba D, Taboada GL, Doallo R, Posada D (2012). jModelTest2: More models, new heuristics and parallel computing. Nat. Methods.

[94] Guindon S, Gascuel O (2003). A simple, fast and accurate algorithm to estimate large phylogenies by maximum likelihood. Syst. Biol..

[95] Rambaut A, Drummond AJ, Xie D, Baele G, Suchard MA (2018). Posterior summarization in Bayesian phylogenetics using tracer 1.7. Syst. Biol..

[96] Rambaut, A. *FigTree, a graphical viewer of phylogenetic trees.* http://tree.bio.ed.ac.uk/software/figtree (2007).

[97] Rozas J (2017). DnaSp6: DNA sequence polymorphism analysis of large data sets. Mol. Biol. Evol..

[98] Wayne D, Poon AFY, Frost SDW, Kosakovsky Pond SL (2010). Datamonkey 2010: A suite of phylogenetic analysis tools for evolutionary biology. Bioinformatics.

[99] Weaver S (2018). Datamonkey 2.0: A modern web application for characterizing selective and other evolutionary processes. Mol. Biol. Evol..

[100] Nielsen R (2005). Molecular signatures of natural selection. Annu. Rev. Genet..

[101] Shastry BS, Komar A (2009). SNPs: Impact on gene function and phenotype. Single Nucleotide Polymorphisms.

[102] Yang Z, Nielsen R (1998). Synonymous and nonsynonymous rate variation in nuclear genes of mammals. J. Mol. Evol..

[103] R Core Team. *R: A language and environment for statistical computing* (R Foundation for Statistical Computing, Vienna, Austria, 2021) https://www.R-project.org/. R version 4.2.2 (2022-10-31).

[104] R Studio Team. *RStudio: Integrated Development for R.* (RStudio, PBC, Boston, MA, 2020) http://www.rstudio.com/R Core Team (2021).

[105] Vihtakari, M. ggOceanMaps: Plot Data on Oceanographic Maps using 'ggplot2'. R package version 2.1.12, https://mikkovihtakari.github.io/ggOceanMaps/ (2023).

