# Supplementary material for: Ancient reindeer mitogenomes reveal island-hopping colonisation of the Arctic archipelagos
Source: Sci Rep. 2024 Feb 20;14:4143. doi: 10.1038/s41598-024-54296-2 (PMC10876933; doi:10.1038/s41598-024-54296-2)
Supplement: Supplementary file 1 — Supplementary Figures. [file 41598_2024_54296_MOESM1_ESM.pdf]

Ancient reindeer mitogenomes reveal island-hopping colonisation of the Arctic archipelagos

Katharina Hold, Edana Lord, Jaelle C. Brealey, Mathilde Le Moullec, Vanessa C. Bieker, Martin R. Ellegaard, Jacob A. Rasmussen, Fabian L. Kellner, Katerina Guschanski, Glenn Yannic, Knut H. Røed, Brage B. Hansen, Love Dalén, Michael D. Martin, Nicolas Dussex

Supplementary Information

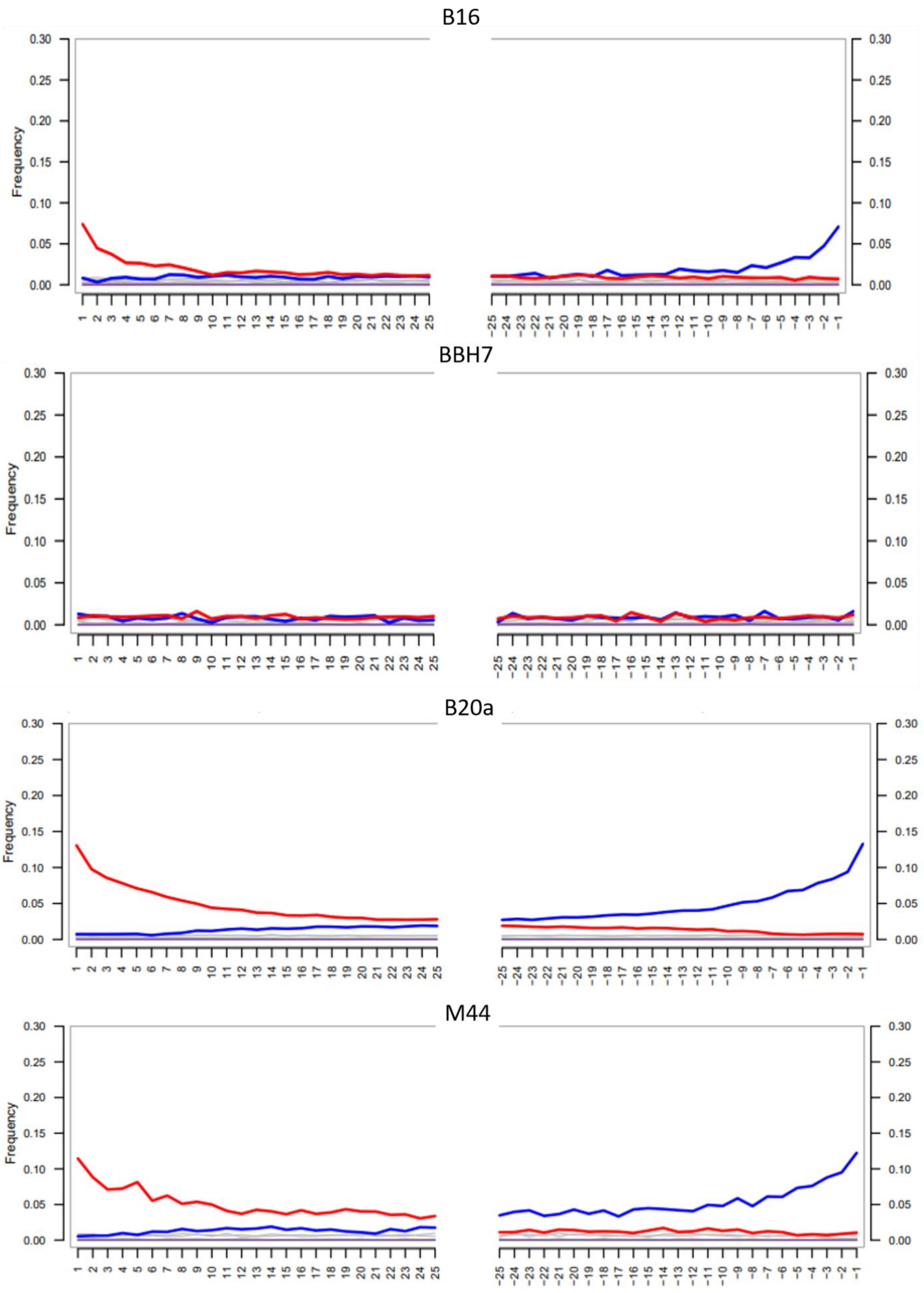

Figure S1: DNA deamination plots of ancient samples B16, BBH7, B20a and M44 using mapDamage v.2.2.1. Frequency of base-misincorporation relative to the distance from the 3’(left) or the 5’(right) end regions. The plots employ colour codes as follows: red= C to T substitutions, blue= G to A substitutions and light grey= all other substitutions.

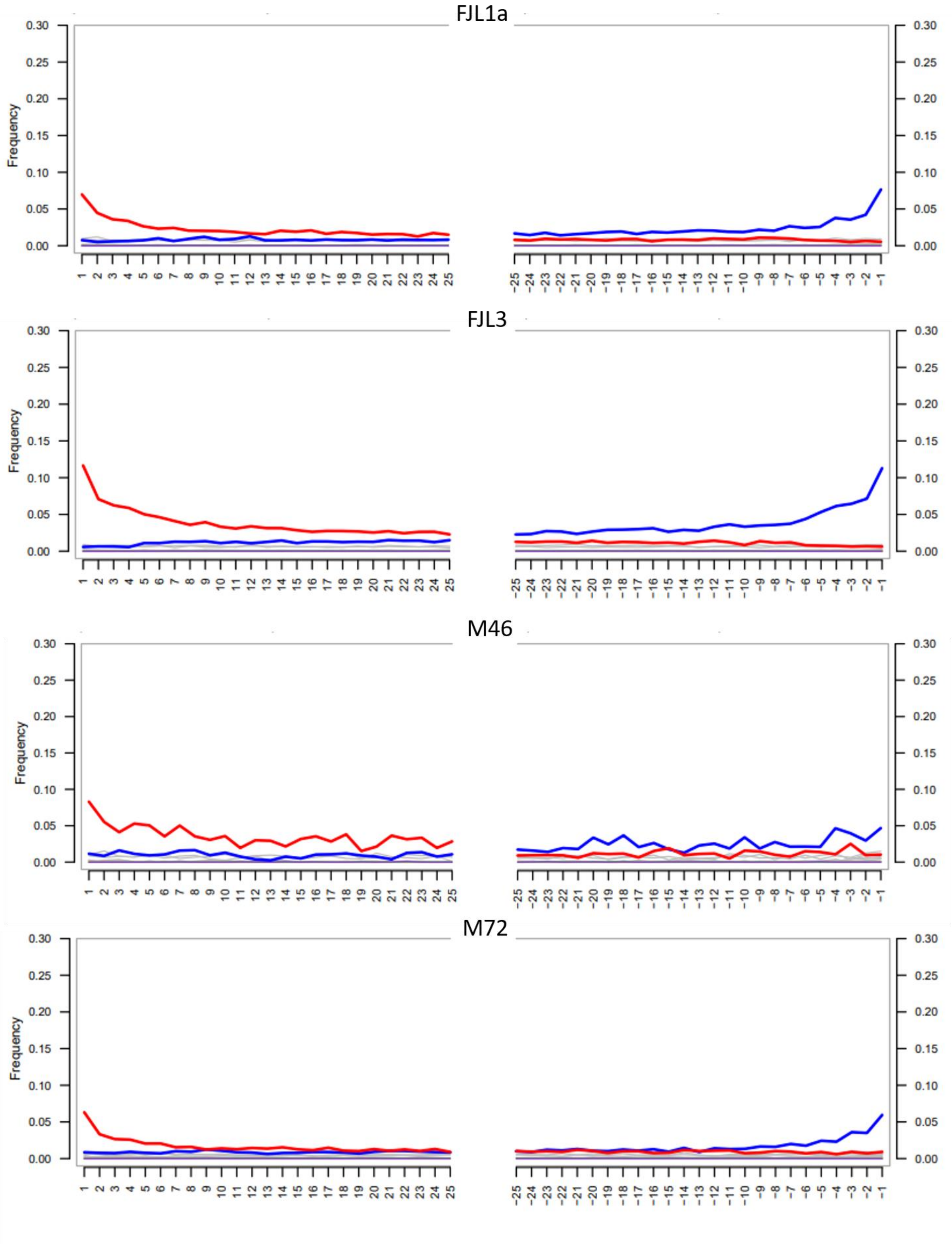

Figure S2: DNA deamination plots of ancient samples FJL1a, FJL3, M46 and M72 using mapDamage v.2.2.1. Frequency of base-misincorporation relative to the distance from the 3’(left) or the 5’(right) end regions. The plots employ colour codes as follows: red= C to T substitutions, blue= G to A substitutions and light grey= all other substitutions.

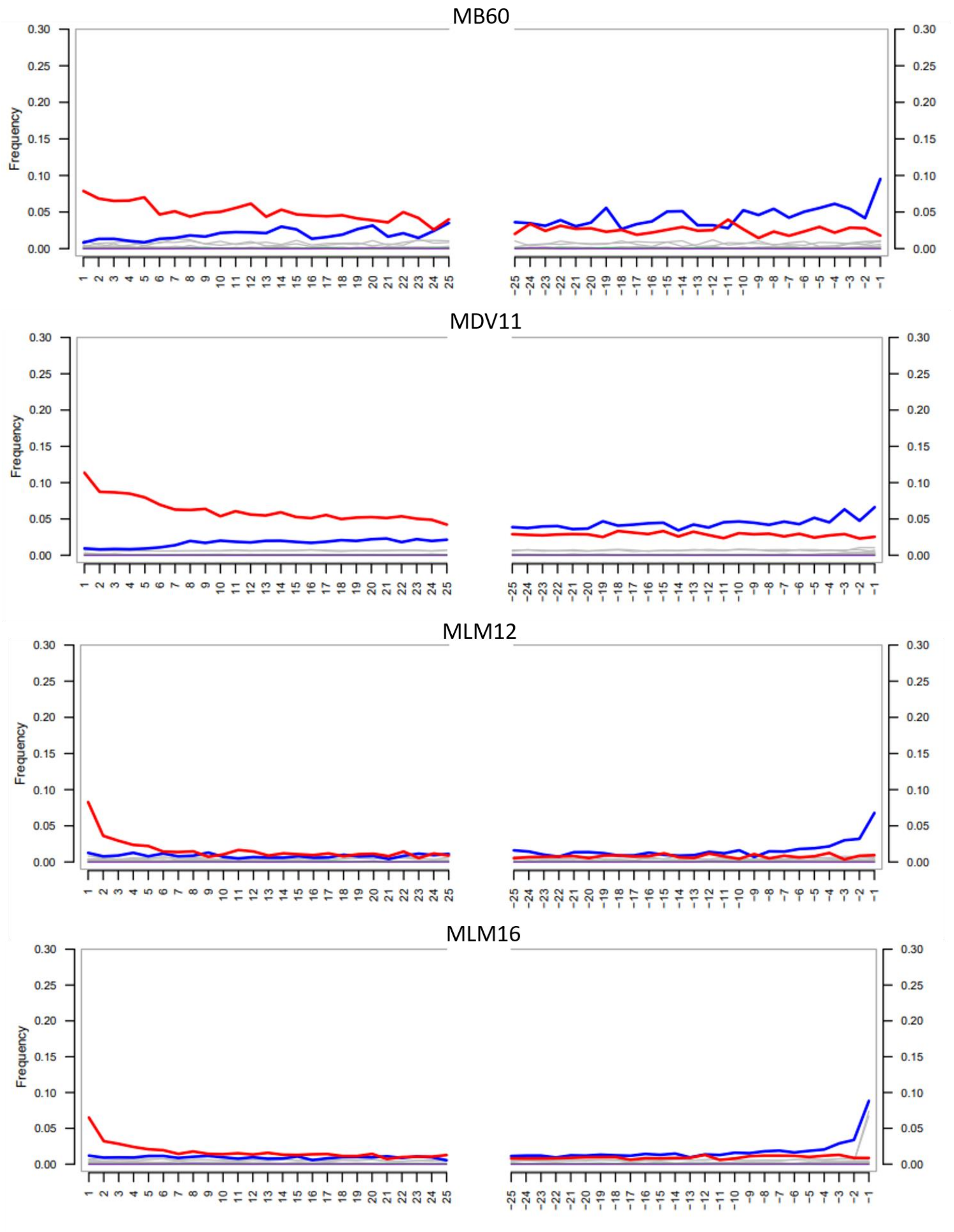

Figure S3: DNA deamination plots of ancient samples MB60, MDV11, MLM12 and MLM16 using mapDamage v.2.2.1. Frequency of base-misincorporation relative to the distance from the 3’(left) or the 5’(right) end regions. The plots employ colour codes as follows: red= C to T substitutions, blue= G to A substitutions and light grey= all other substitutions.

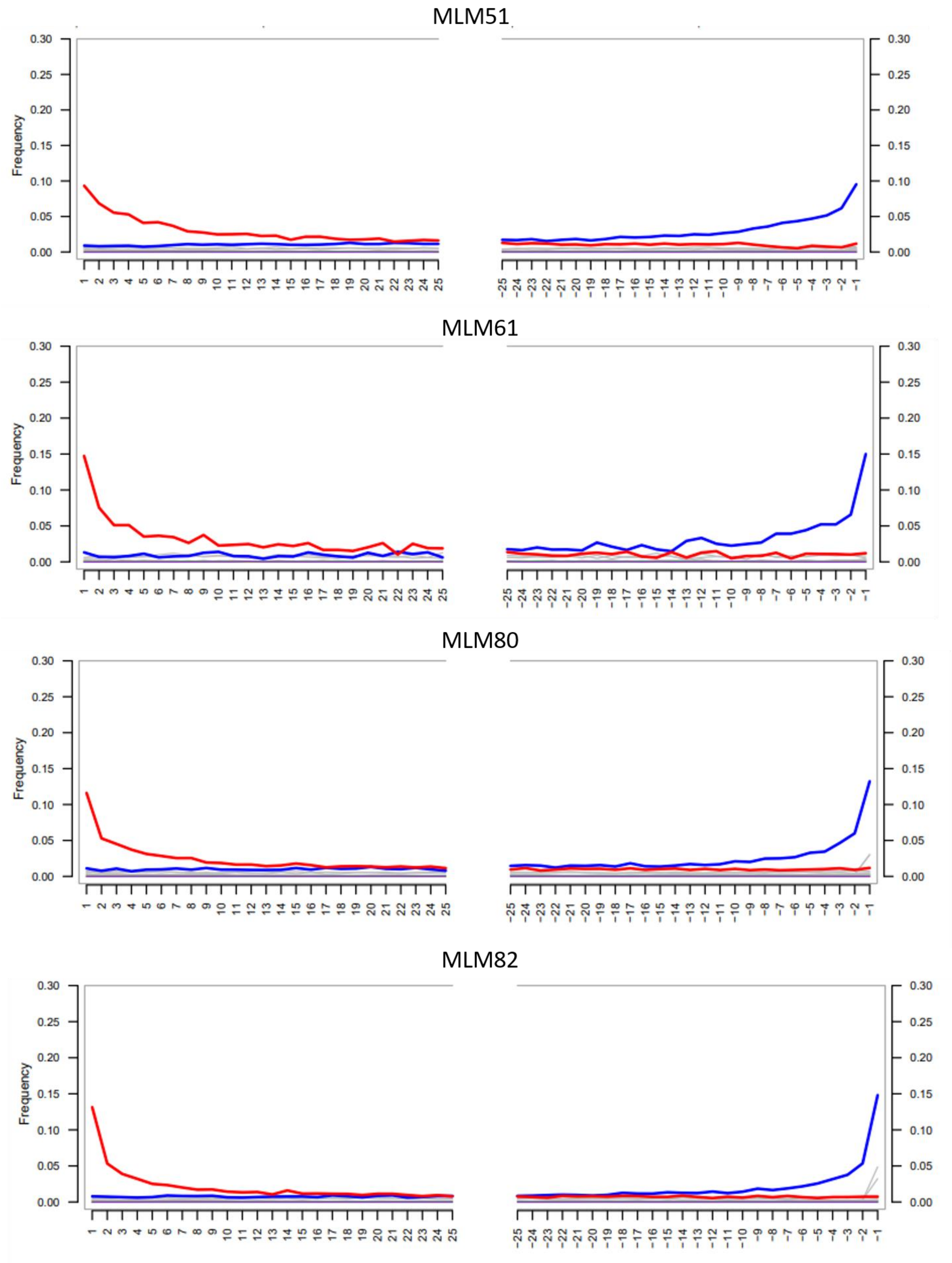

Figure S4: DNA deamination plots of ancient samples MLM51, MLM61, MLM80 and MLM82 using mapDamage v.2.2.1. Frequency of base-misincorporation relative to the distance from the 3’(left) or the 5’(right) end regions. The plots employ colour codes as follows: red= C to T substitutions, blue= G to A substitutions and light grey= all other substitutions.
